# Subcritical and Supercritical Fluids to Valorize Industrial Fruit and Vegetable Waste

**DOI:** 10.3390/foods12122417

**Published:** 2023-06-20

**Authors:** Muhammad Talha Afraz, Xindong Xu, Muhammad Adil, Muhammad Faisal Manzoor, Xin-An Zeng, Zhong Han, Rana Muhammad Aadil

**Affiliations:** 1 School of Food Science and Engineering, South China University of Technology, Guangzhou 510641, China; m.talha.afraz@gmail.com (M.T.A.); fexuxindong@mail.scut.edu.cn (X.X.); adil6200167@outlook.com (M.A.); faisaluos26@gmail.com (M.F.M.); 2 Yangjiang Research Institute, South China University of Technology, Yangjiang 529500, China; xazeng@scut.edu.cn; 3 Guangdong Provincial Key Laboratory of Intelligent Food Manufacturing, Foshan University, Foshan 528225, China; 4 School of Food Science and Engineering, Foshan University, Foshan 528225, China; 5 Overseas Expertise Introduction Center for Discipline Innovation of Food Nutrition and Human Health (111 Center), Guangzhou 510641, China; 6 National Institute of Food Science and Technology, University of Agriculture, Faisalabad 38000, Pakistan

**Keywords:** supercritical fluids, subcritical fluids, valorization, separation, extraction, value-added products, green solvents

## Abstract

The valorization of industrial fruit and vegetable waste has gained significant attention due to the environmental concerns and economic opportunities associated with its effective utilization. This review article comprehensively discusses the application of subcritical and supercritical fluid technologies in the valorization process, highlighting the potential benefits of these advanced extraction techniques for the recovery of bioactive compounds and unconventional oils from waste materials. Novel pressurized fluid extraction techniques offer significant advantages over conventional methods, enabling effective and sustainable processes that contribute to greener production in the global manufacturing sector. Recovered bio-extract compounds can be used to uplift the nutritional profile of other food products and determine their application in the food, pharmaceutical, and nutraceutical industries. Valorization processes also play an important role in coping with the increasing demand for bioactive compounds and natural substitutes. Moreover, the integration of spent material in biorefinery and biorefining processes is also explored in terms of energy generation, such as biofuels or electricity, thus showcasing the potential for a circular economy approach in the management of waste streams. An economic evaluation is presented, detailing the cost analysis and potential barriers in the implementation of these valorization strategies. The article emphasizes the importance of fostering collaboration between academia, industry, and policymakers to enable the widespread adoption of these promising technologies. This, in turn, will contribute to a more sustainable and circular economy, maximizing the potential of fruit and vegetable waste as a source of valuable products.

## 1. Introduction

According to current UN estimates, about 690 million people go to bed hungry every night, accounting for 8.9% of the global population. The hunger-related crisis has been found to be growing in Asia and Africa at a much faster rate than everywhere else in the globe [1]. The excessive production of food in industrialized nations is closely connected to the increased generation of food waste from industrial sources [2]. The Food and Agriculture Organization reported the loss or waste of about one-third of all food produced worldwide, equivalent to about 1.6 billion tons annually [3]. Within this volume, 54% is lost in production, such as post-harvest handling and storage, and the other 46% is lost throughout the supply chain (e.g., processing and distribution), and at the consumer consumption stage. This massive volume of wasted food is not only a severe economic concern but is also an untapped resource in our effort to tackle humanitarian problems. Furthermore, industrial food pollution is a major concern as it involves the consumption of an excessive amount of raw material and produces more effluents and solid waste [4].

Food industries are now moving towards a circular economy as it offers significant economic gains in terms of reduced per-unit cost and the potential to generate additional income streams for the industry [5]. It is also considered a step towards sustainable manufacturing and environmental protection by integrating valorization strategies. However, current value addition and disposal practices include landfilling, organic fertilizer, animal feed components, open burning, and incineration, which are costly and harmful to the environment [6]. Therefore, there is a need to develop new techniques for recovering valuable compounds, which can be converted or utilized in an environmentally friendly way [7]. In this context, valorizing industrial fruit and vegetable waste (FVW) using novel extraction techniques is a useful approach [8].

Novel technologies for the extraction of nutrients and bioactive compounds have received considerable attention, and among these, critical fluid extraction technology stands out due to its operational simplicity, low extraction temperature, and non-polluting characteristics [9]. It has found broad application in the extraction of diverse nutrients and bioactive compounds from an array of food processing byproducts. Notably, it has been employed to extract polyunsaturated fatty acids from apple [10], avocado [11], guava [12], plum [13], and passion fruit [14] byproducts; carotenoids from vegetable and fruit peels [15,16,17]; and polyphenol antioxidant bioactive substances from fruit processing byproducts [15,18,19,20]. SFE is versatile in its utility, not merely as a standalone method but also in conjunction with other extraction technologies. Its combination with mechanical expression has been employed to extract phenolic substances and oils from olive kernels. This synergetic approach not only enhances extraction efficiency but also serves to preserve the biological activity of the extracted substances [21].

Critical fluid extraction techniques (supercritical and subcritical) are good alternatives, if ”generally recognized as safe” (GRAS) fluids are applied in the process [22]. These techniques are environmentally friendly and provide auxiliary benefits such as solvent-free bio-extract, no required post-extraction treatment, downstream processing, reduced extraction time, and high extraction yield [22]. Furthermore, increased fractionation and product selectivity at the point of collection or extraction using SC-CO_2_ extraction significantly eliminate the need for additional resources or energy-intensive purification stages [23]. Compared to ordinary extraction, SC-CO_2_ extraction is cleaner and more efficient due to the absence of residual solvents in the extracted materials. Therefore, valorization of industrial FVW using critical fluid extraction technologies offers a beneficial solution, compared to low value-added practices such as dumping in landfills, using FVW fertilizer or animal feed, or incineration [24].

In evaluating the attributes of extracts recovered through supercritical fluid (SCF) extraction, it is imperative to consider not only the physical and chemical properties but also the consequential implications for human health. This is critical as the primary goal of these extractions is often their integration into functional foods. The efficacy of SFE technology transcends improving extraction efficiency. It also plays a pivotal role in preserving the bioactivity of recovered substances and enhancing the functional attributes of the extract. Given the low-temperature extraction conditions of supercritical fluids, this technique is highly compatible with the recovery of biologically active compounds from agri-food byproducts, particularly thermolabile or easily oxidizable molecules [25]. Recent study highlights the superiority of supercritical CO_2_ (alone) extraction for achieving high contents of α-tocopherols and γ-tocopherols from tomato waste (seeds and skin) extract, surpassing SC-CO_2_ + ethanol-based extraction [16]. Similarly, other studies corroborate that the deployment of SFE technology can augment the extraction efficiency of bioactive compounds while concurrently preserving its antioxidant activity [26]. Other publications have also reported the significance of this technology in extracting valuable compounds [25,27,28,29,30].

This review aimed to collect the existing knowledge and evaluate the valorization potential of industrial FVW and the potential economic stream generation. Moreover, the intended utilization of bio-extracts in the food and pharmaceutical industries is also reported.

## 2. Wastes Generated by the Major Food Industries

**Figure 1:**
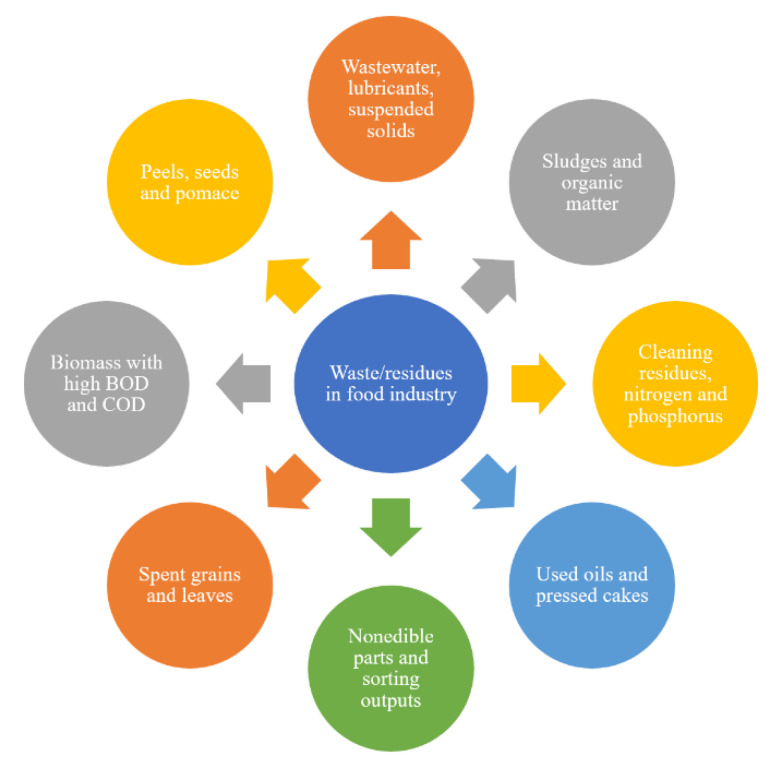
Effluents and solid waste generated by the food industry.

Industrial food waste can be divided into two broad terms: effluents and solid waste. Figure 1 shows the food industry’s major effluent and solid waste. Cleaning water, pits, peels, seeds, pulp, rag (membranes and cores), and other non-edible components (discolored, rotted or damaged sections, bruised and over-ripe portions) are among the waste created by the processing of raw food commodities in the fruits and vegetables industry [8]. In the grain milling industry, the industrial waste may include the water used in the cleaning, tempering, and conditioning process, lubricants, suspended solids (husk, broken and diseased grains), and organic matter in the wastewater [31,32]. In the dairy industry, suspended solids and organic matter with high biological oxygen demand and chemical oxygen demand, cleaning residues, nitrogen, and phosphorus, a huge amount of wastewater, oil, and grease, and whey concentrate represent the main components of the waste [4]. Used oils, cake or meal, seeds, and pomace are found in the fat and oil industry [33]. Non-edible parts, wastewater, bones, and excessive fat are the major components of waste generated in the meat industry.

Wastewater is the major effluent in most food industries and contains suspended solids and organic matter. Moreover, it also contains various cleaning and sterilizing agents which are used for the cleaning of utensils and equipment. Solids or semi-solids found in wastewater effluent may constitute rind, spent coffee and tea, spent grains (brewing industry), bones, and pomace. The dairy and fruit and vegetable processing industry remain the highest wastewater effluent emitter as they generate 6–10 L of wastewater per liter of processed milk and 1–17 m^3^ per ton (1–17 L per kilogram) of processed fruits (citrus), respectively [4,34,35]. Moreover, weight, size, shape, and color sorting, a requirement in premium products, also generate a huge amount of organic waste.

Industrial FVWs are sources of valuable compounds such as fibers, minerals, sugars, vitamins, aromatic compounds, phenolic compounds, and carotenoids [36]. Most of these compounds are synthesized in the secondary metabolism of plants to provide protection against the environment [37]. In human nutrition, they are considered bioactive compounds responsible for anti-inflammatory, antioxidant, anticancer, neurosedative, and antiviral activities, among others [38]. The beverage processing industries produce 20–60% of waste/byproducts [8]. From the environmental protection point of view, this generation of waste/byproducts must be reduced by different technological approaches for the complete utilization and extraction of valuable compounds or ingredients [8]. The processing of fruits and vegetables generates a large amount of waste in terms of seeds, skin, and pomace, after the removal of the pulp. The seeds contain about 15–18% proteins, 28–31% oils, 10–12% carbohydrates, and 3.2–17% crude fiber, and all these components are exploitable [39,40].

Both the peel and the flesh of fruit and the vegetable processing waste are characterized by a high content of dietary fibers (soluble and insoluble); however, the pomace of most fruits and vegetables represents the highest content of insoluble dietary fibers. The fruit’s pulp also contains a considerable amount (almost 78%) of dietary fibers [8]. These biomolecules in fruit and vegetable processing waste can be valorized in the fortification of the fiber fraction and the production of various nutraceuticals [41]. These are non-starch polysaccharides, including other components such as resistant dextrins, inulin, beta-glucans, lignins, pectins, cellulose, oligosaccharides, and resistant starch [42,43]. Dietary fibers will also impart health-promoting benefits, such as the lowering of cholesterol and glucose, due to their swelling and water-holding capacity [44]. 

Seeds and kernels as processing waste can be manipulated and used as a potential source of unconventional oils with biologically active compounds [45]. Similarly, waste materials can also be maneuvered to produce flavors/essential oils by applying different treatments. They are loaded with various health benefits such as anti-inflammatory, antimutagenic, anticancer, antifungal, antiviral, vermicide, and antibacterial activities [46]. The valorization of important biomolecules from waste/byproducts reduces environmental pollution, generates additional income, and provides a secondary advantage for new enterprises dealing with food processing [47].

Since ancient times, mankind has benefited from phytochemicals having bioactive properties. These may include anti-inflammatory, antimutagenic, antioxidant, anticancer, antithrombotic, antibacterial, antiviral, and vasodilator properties [48]. The market demand for bioactive compounds is increasing due to changing lifestyles with customers leaning towards functional foods because of their health benefits. The global functional foods market was valued at USD 27 billion in 2020, and it may rise to USD 51.71 billion by 2025 [49]. Utilization of valorized compounds in different industries is summarized in Table 1.

## 3. Subcritical and Supercritical Fluid Technology in Food Processing

**Figure 2:**
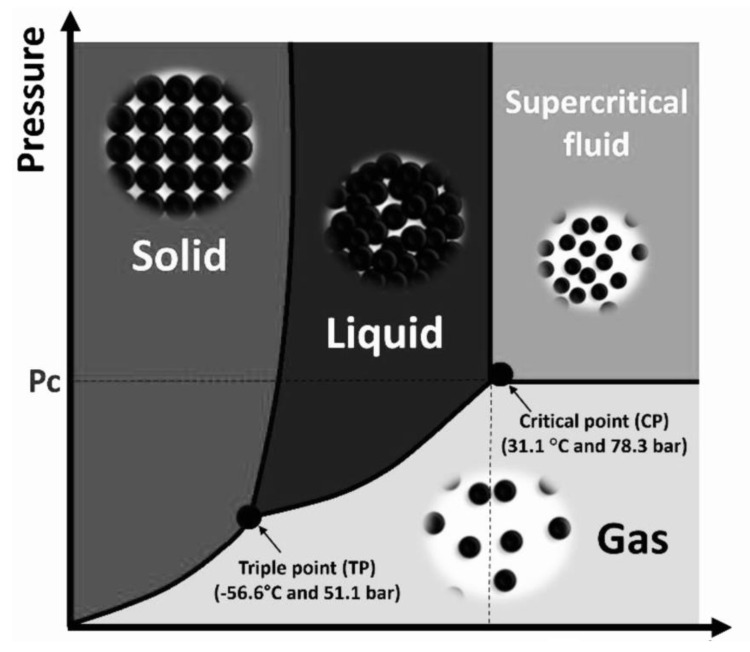
SCFs with both liquid and gas properties [68].

Supercritical fluid (SCF) is a unique state of matter that exhibits both gaseous and liquid properties once it surpasses its critical temperature and pressure (Figure 2). This state imbues the fluid with characteristics that are particularly advantageous for extraction purposes. Specifically, supercritical fluids demonstrate lower viscosity and higher diffusivity compared to their liquid or gaseous states, thereby enhancing their ability to penetrate solid matrices and consequently increasing extraction yields [60,61]. Moreover, the density of supercritical fluids, a property integral to the extraction process, can be fine-tuned by adjusting the pressure and temperature conditions. This adaptability allows for targeted extraction of specific compounds, thereby enhancing the selectivity and efficiency of the process [62]. Among the various supercritical fluids, carbon dioxide (CO_2_) is the most commonly employed in food-related applications. Its popularity is due to several reasons, e.g., CO_2_ is a versatile solvent, capable of extracting a wide array of bioactive compounds, and it is readily available, making it a practical choice. Lastly, and perhaps most importantly from a food safety perspective, CO_2_ leaves no residues in the extract, ensuring the purity of the final product [24]. In addition to the aforementioned advantages, CO_2_ boasts several other qualities that make it an ideal choice for use as a supercritical fluid. It is relatively inert, non-flammable, and non-toxic, which contributes to the safety of the extraction process. Furthermore, CO_2_ is economically viable due to its inexpensive nature and the fact that it is recyclable. From a health perspective, it is considered harmless to humans [63]. With critical points at 31.1 °C and 7.38 MPa, CO_2_ can be used under conditions that are particularly conducive for extracting a wide array of volatile and heat-sensitive compounds, thereby preserving their functional and nutritional properties [64,65,66]. When compared to traditional solvents used in food processing, such as dichloromethane and hexane, CO_2_ is significantly superior in terms of safety, toxicity, operating costs, and regulatory compliance [64]. SCF technology has found applications in numerous waste valorization processes (as illustrated in Table 1). SCF-CO_2_ has proven particularly effective for extracting non-polar and moderately polar compounds. However, its lower polarity does present a limitation when it comes to extracting polar compounds. This challenge can be mitigated by optimizing the SCF extraction process with the use of a co-solvent, thereby extending the range of compounds that can be efficiently extracted [61]. Polar compounds, e.g., polyphenols, can be extracted using SCFs in combination with co-solvents (methanol, ethanol, and acetone) as they enhance the solvating power, solubility, and extractability of polar compounds [67].

The first commercially successful application of supercritical fluid technology was established by Hag A.G. for decaffeinating green coffee beans in Bremen, Germany, in 1978 and by Carlton and United Breweries for the extraction of hop flavor in Melbourne, Australia, in 1980 [69]. These developments opened the door to vigorous research and development in this newly developed processing/extraction technique. SCF technology is non-conventional, green, cost-effective (with down processing), and energy-efficient, with negligible environmental effects [64]. Therefore, it is an advantageous alternative to conventional extraction techniques, which require expensive solvents, a laborious procedure, and high energy consumption and create an adverse impact on the environment [70]. 

**Figure 3:**
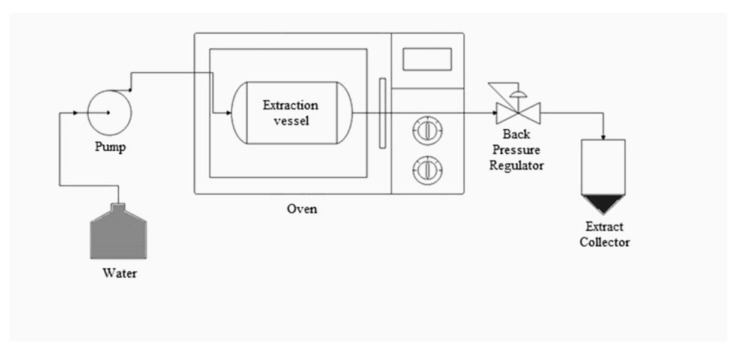
Schematic diagram of SWE [78].

Alongside supercritical fluid (SCF) technology, subcritical fluid technology (SubCF) also presents an environmentally friendly approach for the extraction of industrially significant bioactive compounds. In SubCF, water is predominantly used as the extraction solvent for food-related applications. However, other solvents such as propane [71], n-butane [72,73], ethanol [74], and methanol [75] have also been employed. Subcritical water extraction (SWE) is a specific method within SubCF which utilizes water that is maintained at conditions between its boiling point and critical point temperature (100–374 °C). Figure 3 illustrates the schematic diagram of SWE. The pressure is adjusted to ensure that the water remains in a liquid state [76]. The temperature and pressure ranges typically used for SWE are 100–250 °C and 1–8 MPa, respectively [22]. The underlying principle of SWE is that by increasing the water’s temperature while maintaining high pressure, the water remains in a liquid state (for instance, at 250 °C and 50 bar) and exhibits a reduced dielectric constant. This change in dielectric constant effectively alters the polarity of water, thereby enhancing its solvating power and enabling the efficient extraction of a broad range of compounds. The unique behavior of subcritical water stems from its ability to weaken hydrogen bonds and other intermolecular forces among water molecules. This weakening leads to a substantial increase in the extraction rate under subcritical conditions. As the dielectric constant of water drops (ε = 27) under these conditions, changes in physical properties such as decreased viscosity and surface tension, along with increased diffusivity, occur. This transformation results in water behaving more like an organic solvent, equipping it with the ability to dissolve low to moderately polar analytes [77]. Further contributing to subcritical water’s enhanced extractability is the correlation between water temperature and the average kinetic energy of the molecules. As water temperature increases, the average kinetic energy of the molecules also elevates, leading to the disruption of bonds within the water molecules. Moreover, the extraction rate and selectivity of analytes can be adjusted by manipulating the temperature and pressure conditions of subcritical water. However, a thorough understanding of how subcritical water’s properties change with variations in temperature and pressure is essential for effectively applying this technology [22].

## 4. Recovery of Bioactive Compounds

Subcritical and supercritical fluid (SCF) technologies have been used novel technologies in the extraction of valuable compounds from different food materials [12,19,40]. Liu et al. [40] reported the application of supercritical CO_2_ to extract volatile and non-volatile components from custard apple (*Annona Squamosa*) seed powder. The researchers reported that optimized conditions for the extraction of volatile and non-volatile components were 15 MPa at 308 K with a flow rate of 1.5 mL/min and 25 MPa at 318 K temperature and 2.5 mL/min, respectively. The extracted components can be utilized in the flavoring industry. Moreover, the antifungal and antibacterial properties of the extract have also been reported in this study. In another study, supercritical CO_2_ was used to extract oleoresins from tomato (*Lycopersicon esculentum* L.) waste (seeds and skins) and provided a yield of 12.5–12.9%, while using the dichloromethane extraction method as a control. Additionally, the effect of ethanol as a co-solvent was also evaluated. The extract obtained using supercritical CO_2_ (150 bar, 20 °C, and 5 mL/min) without ethanol possessed the highest content of α-tocopherols (80 mg) and γ-tocopherols (575 mg/100 g oleoresin), while lycopene and β-carotene contents were 205 and 75 mg per 100 g of extracted oleoresin, respectively. The highest content of polyphenols (9305 mg GAE/100 g of oleoresin) was reported using ethanol (10%) as a co-solvent. Moreover, the synergistic effect of tocopherols, carotenoids, and polyphenols enhanced the antioxidant activity of all extracted oleoresins. These oleoresins can also be used in various food and pharmaceutical formulations [16].

In another study, it was concluded that supercritical CO_2_ (380 bar, 80 °C, and 15 kg/h) used with exogenous tomato oil from the co-solvent tank can be utilized to recover 99.3% of extractable lycopene (502 μg/g of seed oil) from tomato solid waste [79]. Moreover, highly moist (102.7 g/kg) tomato pomace allowed 97% lycopene recovery using supercritical CO_2_ technology [57]. Similarly, the processing waste (pomace, skin, and seeds) of kiwifruit has been utilized for the extraction of polyphenolic compounds with antioxidant activities. Extraction under 50 bar, 200 °C, and 90 min has been reported to provide the highest concentration of phenolic compounds (60.53 mg CaE/g DW), and chlorogenic acid, caffeic acid, catechin, p-coumaric acid, and protocatechuic acid were the most abundant compounds in the extract [54].

The grape (*Vitis vinifera* L.) winemaking industry generates a considerable amount of solid waste (skin, seed, and pomace). It can be processed for the extraction of highly valuable compounds, such as antioxidants, vitamins, and polyphenols, as grape skin and seeds contain about 90–95% polyphenols [80]. SFE-CO_2_ has been applied to extract these compounds (trans-resveratrol, β-sitosterol, α-tocopherol, and ascorbic acid), and a solid–liquid extraction technique was utilized as a control [19]. It was reported that SFE-CO_2_ under optimized conditions (250 bar, 60 °C, 2 mL/min CO_2_, and 0.4 mL/min ethanol as co-solvent) recovered higher concentrations of all considered analytes as compared to the control except for α-tocopherol and β-sitosterol from seeds as these analytes might require a second cycle of extraction. These extracted compounds found their application as an economic component of the pharmaceutical and nutraceutical matrix [19]. SFE technology was applied for the valorization of broccoli (*Brassica oleracea* var. italica) byproducts (stems and leaves) using ethanol and CO_2_ as solvents [18]. The extract was rich in β-carotene, chlorophylls, phytosterols, and phenolic compounds. The authors reported that the maximum concentration of β-carotene was extracted at 443 bar, 40 °C, 31 g/min flow rate, 7% ethanol as co-solvent, and 68 min. Moreover, the extract also exhibited higher antioxidant activity as compared to the conventional extraction and the former extract also showed a cytoprotective effect. The extract has potential use as a cosmeceutical ingredient [18].

Viti viniculture practices generate a large amount of pruning waste, which can be valorized for valuable compounds [55]. The potential of pruned vine shoots of two grape varieties (*Tinta Roriz* and *Touriga Nacional*) for the extraction of phenolic compounds was studied. The potential of SWE (40 bars, 150 °C, 40 min), microwave-assisted extraction (MAE; 100 °C, 20 min, ethanol/water 60:40 *v*/*v*), and conventional extraction (55 °C, 2 h, ethanol/water 50:50 *v*/*v*, 100 rpm) was evaluated for the extraction of bioactive compounds and functional properties of the extract. It was reported that all extracts showed antimicrobial (bacteria and yeast) and enzyme inhibition (acetylcholinesterase and α-amylase) activity, thus indicating potential in the treatment of diabetes and Alzheimer’s disease. *Tinta Roriz* variety exhibited higher bioactive potential as compared to *Touriga Nacional*. The highest flavonoid content (18.7 ± 1.2 mg EE/g DW) was reported in the extract obtained by SWE, and antioxidant activity assessed by DPPH and FRAP assay was highest for SWE, followed by MAE and conventional extraction. However, the highest phenolic content (32.1 ± 0.9 mg GAE/g DW) was recorded in MAE. The concentration of bioactive compounds depended on several factors: age, variety, and environmental conditions [81].

Other studies have also reported the extraction of bioactive compounds from fruit and vegetable processing waste by using subcritical and supercritical extraction technology, such as olive leaves [82], mandarin (*Citrus Unshiu*) peel [83], orange peel [84,85], apple pomace [86], apricot (*Prunus mume*) pits [75], passion fruit rind [87], and grape seed [88]. Table 2 summarizes the bioactive compounds extracted by critical fluids.

## 5. Extraction of Unconventional Oils

The majority of the published literature focusing on the extraction of essential/unconventional oils from fruit and vegetable industrial waste using critical fluid extraction techniques has reported similar or slightly higher yields as compared to conventional techniques [89]. However, critical fluids provide auxiliary benefits in terms of lower extraction time, no residual issues, and environmental friendliness and obviate the use of dangerous solvents, among others. Apple (*Malus pumila*) seed oil was also extracted by using SFE technology. It was reported that the extract from SFE (24 MPa, 40 °C, 1 L/h, and 140 min) had a higher content of linoleic acid (63.76 g/100 g of oil) as compared to the control extraction using the Soxhlet technique (49.03 g/100 g of oil). Despite the high content of unsaturated fatty acids, SFE extract expressed higher oxidative stability (21.4 h) than the controlled extract (12.1 h). Additionally, the most abundant phenolic compound was phloridzin (2.96 μg/g of seed), and the extract was free from anti-nutritional compounds [10]. Similarly, the guava (*Psidium guava*) seed extract by supercritical CO_2_ (35.7 MPa, 52 °C, 30 g/min and 150 min) is also characterized by its higher content in linoleic acid (78.5%, *w*/*w*) followed by oleic acid (13.8%, *w*/*w*). Additionally, the extract has also reported a rich profile of phenolics, tocopherol, and phytosterol compounds [12]. Fractionated linoleic acid can be used in the enrichment of various dairy products. Furthermore, SFE-CO_2_ has been applied to mango waste (kernel) to extract the polar lipids from its lipid matrix. About 20% of the whole fruit constitutes mango kernel, and 7–11% of it contains good-quality fat. The fat content can be further classified as 95–96% non-polar and 3–4% polar lipids [90,91]. The highest extraction yield of polar lipid was 3.28% with desirable phosphorus content (91.2 mg/kg) under optimized conditions of SFE-CO_2_, 50 MPa, 40 °C, and 30 g/min CO_2_ [56]. These valorized lipids have wide applications in the food [90], nutraceutical [92], and pharmaceutical [93] industries. Moreover, in the literature, mango kernel fat has been reported as a substitute for cocoa butter [90,94].

The processing of avocado fruit (*Persea americana*) generates a significant amount (21–30%) of solid waste in terms of seeds, peels, and exhausted pulp, depending on the cultivar [95]. SC-CO_2_ (25 MPa, 80 °C, 1.5:1 ethanol) has been applied on avocado waste (seeds and peels) of Hass cultivars in Mexico and Brazil, for the extraction of oil. Soxhlet extraction (SE) was used as a control and ethanol was used as a solvent and co-solvent in the case of SC-CO_2_. Greater oil extraction yields were recorded in SE 14% than in SC-CO_2_ 6.9%, and the major components of the extracted oils were oleic and linoleic acid regardless of the extraction method. The total phenolic content and antioxidant activity of extracted oil from the seeds and peels of Mexican origin were higher as compared to Brazilian origin, regardless of the extraction method applied. Generally, higher extraction yields were recorded in SE than SC-CO_2_ due to the longer extraction times and higher number of solvents used, thus greater solvent and energy consumption. SC-CO_2_, on the other hand, is more advantageous in terms of lower capital cost and minimal environmental impact [11]. Similarly, *Prunus domestica* L. is a widely cultivated fruit in Europe and is often regarded as the European plum. Its processing generates a considerable amount of solid waste (plum kernel), thus creating an opportunity to convert the waste stream into an economic stream. Cold pressing (20 Hz, 40 °C) and SC-CO_2_ (300 bar, 40 °C, 2 kg/h flow rate) have been applied to extract unconventional oil, which is characterized by essential FAs and tocopherols. Both extracts were rich in oleic acid content; however, the highest content was reported in the SC-CO_2_ extract (68.66%), and linoleic acid was the second most abundant content (22.24%). SC-CO_2_ extract also leads to 4–5.8 times higher tocopherol content than cold-pressing extract. This alternative oil source found its potential application in the food and pharmaceutical industries due to low saturated FAs and amygdalin content [13].

Campalani et al. [50] reported SC-CO_2_ (300 bar, 70 °C, 5 h) extraction of purer lipophilic compounds from the peels and seeds of blueberry, wild strawberry, pomegranate, raspberry, blackcurrant, and blackberry. Strawberry pomace extract was 26% by weight; the extract was rich in essential FAs, of which 46.8 mg/mL was saturated FAs, 64.0 mg/mL monounsaturated FAs, and 145.8 mg/mL polyunsaturated FAs. The percentage of extracted FAs was greater than when using conventional solvent (hexane), which is toxic and restricts the usage of the extracted FAs in the cosmetic and food industry. In addition, extra purification unit operations are required. Similarly, SC-CO_2_ (47 MPa, 53 °C, 75 min) together with pressurized liquid extraction (PLE, 10.3 MPa, 50–90 °C, 15–45 min) yielded 73.6 g of rich extract from 100 g of lingonberry (*Vaccinium vitis-idaea* L.) pomace, of which 84% was of polar nature. The lipophilic fraction was rich in linoleic (37.4%) and α-linolenic (43.3%) FAs, with other constituents including anthocyanins and pro-anthocyanidins, 231 mg and 15.9 g per 100 g, respectively, and a significant number of antioxidants [96]. Higher percentages of unsaturated (85.62%) and polyunsaturated (57.90%) FAs have been recovered from *Berberis dasystachya* seeds using the SC-CO_2_ (25 MPa, 59.03 °C, 2.25 SL/min flow rate) extraction method as compared to ether (80 °C, 7 h) extraction in a Soxhlet extractor [97]. The major content of the extract was linoleic and linolenic acid, resembling the study of [96]. 

Pumpkin seed oil expresses a significant number of antioxidants in terms of phenolics and tocopherols; thus, in order to protect the thermally unstable components in the extract, SC-CO_2_ has been applied for the extraction of pumpkin seed oil. The highest extraction yield was 30.7% under optimized conditions: 32,140 kPa, 68.1 °C, and 94.6 min; these three variables showed a synergistic effect in the extraction of pumpkin seed oil [98]. Subcritical fluid extraction technology has been applied to yellow passion fruit (*Passiflora edulis* var. *flavicarpa*) waste (seed) for oil extraction. SubFE using compressed propane under optimized conditions (8 MPa and 30 °C) gave a 24.68% extraction yield. However, higher content of essential linoleic acid (68.99%) was reported at 30 °C and 2 MPa and tocopherol (5.98 mg/100 g of oil) at 60 °C and 2 MPa. Extracted oil also presented an antioxidant activity of 75.12%, determined by the DPPH method [14]. The study indicated that the organic waste of yellow passion fruit can be exploited for the development of functional food ingredients.

## 6. Utilization of Spent Material in Biorefinery and Biorefining Process

The concern surrounding the depletion of fossil fuels has driven the search for alternative and renewable energy sources. The conversion of lignocellulosic biomass, such as fruit and vegetable industrial waste/residues, has emerged as an attractive alternative to the production of fuels to replace fossil fuels. In this context, [99] reported that apple seeds degrade at a lower temperature (lower heating value) in the thermochemical conversion process, after the extraction of valuable compounds by using SC-CO_2_. Thus, the spent apple seeds can later be valorized in a biorefinery. Similarly, biomass (tomato pomace) from tomato cannery can be used in a biorefinery to produce bioenergy. After the extraction of significant oil content (rich in lycopene) from the tomato pomace by using SC-CO_2_, the spent material is then used as feedstock for the biorefinery. The exhausted tomato pomace showed a 64% higher biodegradability than the raw tomato pomace, thus generating an additional income stream [57]. After the recovery of ginger oil using SC-CO_2_ (350 bar, 35 °C, 15 g/min CO_2_, 10% ethanol) from the industrial ginger (*Zingiber officinale*) waste (e.g., herbal medicine and beverages), the spent material was further unitized in a microwave-based biorefinery to produce hydrochar (20–24.5 MJ/kg) and chemically rich bio-oils [100]. 

**Figure 4:**
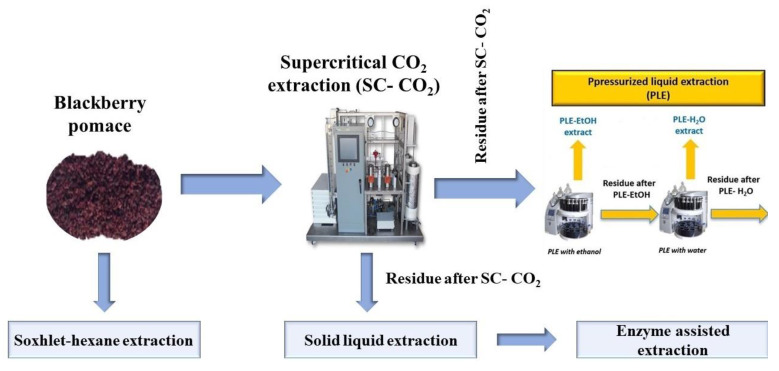
The multistep biorefining process of blackberry pomace [101].

A multistep biorefining strategy was applied to blackberry (*Rubus fruticosus* L.) pomace for the recovery of nutritionally and industrially valuable components (Figure 4). Response surface methodology was applied to optimize the isolation potential of SFE-CO_2_ and pressurized liquid extraction (PLE) techniques. SC-CO_2_ under optimized conditions (54.8 MPa, 64 °C, 171 min) recovered 9.9 g/100 g of a lipophilic fraction rich in healthy polyunsaturated FA (linoleic 64.1%, α-linolenic 12.9%) and monounsaturated FA (oleic 14.5%). Consecutively, the spent residues of SFE-CO_2_ were treated with PLE (10.3 MPa, 90–130 °C, 30–45 min) and recovered ethanol (23.3 g) and water-soluble constituents (5.1 g) under different treatment conditions. Thus, 41.3 g of various useful compounds were recovered from 100 g of blackberry pomace, and 76% of it was polar constituents. This refining strategy reduced the 93% antioxidant capacity of the starting material, showing its efficiency in recovering antioxidants from blackberry pomace. The major anthocyanin was cyanidin-3-glucoside detected in both the starting material and the PLE extract (4.8–551 mg/100 g). Green technologies surpass the efficiency of conventional extraction techniques in terms of solvent consumption, extraction time, total yields, maceration, in vitro antioxidant capacity, and phytochemical composition [101].

A similar biorefining approach utilizing SC-CO_2_ and PLE was utilized for the recovery of lipophilic and polar fractions in blackcurrant (*Ribes nigrum*) pomace [102] and cranberry (*Vaccinium oxycoccos*) pomace [103]. The lipophilic fraction obtained from SC-CO_2_ was rich in polyunsaturated FA in both the fruit pomace and the blackcurrant pomace extract; it was also rich in tocopherols (vitamin E). However, the polar fraction obtained from PLE (and enzyme-assisted extraction in blackcurrant pomace) in each pomace was characterized by its antioxidant capacity. Thus, based on these three studies, it can be concluded that a multi-step biorefining process based on SC-CO_2_ and PLE can be utilized in the “zero waste” processing of blackberry, blackcurrant, and cranberry pomace at the industrial scale. 

## 7. Economic Evaluation/Estimations and Impediments in the Valorization Process

Several research studies have delved into the economic potential of supercritical CO_2_ extraction facilities specifically designed for the valorization of industrial fruit and vegetable waste. Among these, the work of Kayathi and colleagues [56] stands out. They meticulously calculated the comprehensive costs involved in establishing a facility intended for the extraction of extra virgin mango kernel oil. According to their analysis, the capital expenditure to set up a plant with a processing capacity of 3000 tons of mango kernels annually would amount to approximately USD 4953,225. When combined with the yearly operational expenses of around USD 3999,440, the total investment required to kickstart such an industrial-scale extraction venture totals nearly USD 8952,665. While this figure may seem daunting, Kayathi et al. [56] estimate that the initial investment would be recouped within a span of just four years. Further, they project that the net present value of mango kernel oil would be five times higher than the initial investment after a decade of operation. The economic implications of this venture extend beyond the extraction plant itself. It would also open up a valuable revenue stream for mango producers and processors who could sell their residual waste at a rate of USD 3.00 per ton, as factored into the cost analysis of the extraction plant. This innovative approach to waste valorization thus presents an exciting avenue for sustainable and profitable business practices within the fruit and vegetable industry.

Another example comes from the processing of tomato cannery waste, specifically tomato pomace, after lycopene extraction. By integrating supercritical CO_2_ extraction with an anaerobic digestion-based biorefinery, the pomace was further transformed into bioenergy and fertilizers. This ingenious process gave the spent tomato pomace, previously valued at EUR 10/ton, a new worth, generating an additional income of EUR 787.9/ton [57]. One of the most effective strategies for reducing the extraction costs of valuable compounds from fruit and vegetable waste is to enhance downstream processing and modify the nature of the feedstock. For instance, the extraction cost of wax from milled date palm leaves using supercritical CO_2_ was estimated to be EUR 13.62/kg [104]. However, a simple change in the feedstock format, using pelletized leaves, reduced the cost significantly to EUR 8.55/kg. This reduction was due to the increased density and loading amount of biomass. Further cost reduction was achieved by utilizing the post-extraction material for energy generation, specifically burning it for electricity production. This step dropped the cost even further to EUR 3.78/kg. Although burning the post-extraction material is considered a low value-added step compared to more advanced processes such as hydrolysis, fermentation, and pyrolysis, it is a testament to the potential of waste valorization. In addition, the extraction time, which correlates directly with labor and operating costs, can be managed more efficiently by reusing or recycling utilities [22]. This reflects the intricate balance between process optimization, resource management, and economic viability in utilizing supercritical fluids for waste valorization.

While SFE technology is still in its developmental stages, and real-time applications on an industrial scale are relatively rare, researchers are turning to software tools such as SuperPro Designer to estimate manufacturing costs and other economic parameters [105,106,107]. One such study by Restrepo-Serna and Alzate [108] employed such a model to evaluate the economic feasibility of extracting oil from yellow passion fruit seeds using SFE. According to their model, this process could achieve an impressive profit margin of 86.94% with a flow rate of 100 kg/h, a production cost of 2.09 USD/kg, and a payback period as short as 0.45 years. The capital cost, inclusive of the equipment, was estimated at USD 0.34 million. Economic evaluation indicated that manufacturing costs could be significantly reduced by scaling up production [109]. In this regard, another study reported the extraction of polyphenols and capsiate-rich oleoresin from biquinho pepper at a 500 L scale using a combination of supercritical fluid and pressurized liquid extractions (SFE + PLE). The study found that the combined process yielded a lower cost of manufacturing (5316.41 USD/kg) compared to executing the processes separately (7422.20 USD/kg) [105]. These studies suggest that with strategic planning, economic modeling, and scale optimization, SFE can become a cost-effective solution for extracting valuable compounds from plant-based materials.

Despite the promising potential of SFE, it is important to note that not all product opportunities have been industrially exploited. In fact, there is a distinct lag between research advancements and their translation into industrial applications. This delay arises primarily due to the numerous validations that are necessary before a new technology can be industrially adopted. Firstly, the efficiency of the technology needs to be convincingly demonstrated. This is not a trivial task, as it involves not just proving that the technology works in a laboratory setting but that it can also perform reliably and cost-effectively at an industrial scale. After that, products derived from SFE must be included in novel food regulations. This often involves rigorous testing and lengthy regulatory processes to ensure that the products are safe for human consumption. Moreover, the extraction process itself and the resultant products must be validated for their nutritional profile, toxicity, allergenic potential, and the presence of contaminants. These checks are crucial to ensuring the safety and quality of the products, but they can be time-consuming and costly [110].

Certain other obstacles currently limit the widespread commercial acceptance of supercritical fluid extraction technology. A key issue is that supercritical CO_2_ extraction is typically a batch process and lacks the necessary standardization for broader industrial applications [111]. However, many companies are investigating the feasibility of transforming it into a continuous process, a shift that would significantly broaden its potential applications. The perception of high costs associated with this technology is another significant barrier. This perception is fueled by the necessity of maintaining high-pressure conditions to achieve a supercritical state and the substantial initial investment and integration costs [23]. However, recent cost analyses of supercritical CO_2_ technology have shown promising economic viability for recovering nutritionally, pharmaceutically, and industrially valuable compounds from fruit and vegetable industrial waste, provided certain conditions are met [56]. These conditions include the physical state of the waste (whether it is moist, dried, milled, or pelletized), the specific material chosen for extraction (such as pomace, seeds, and peel), and the intended use of the extracted compounds. While the high capital costs can deter smaller processing companies from adopting this technology, continuous engineering improvements and innovative uses of spent material can further mitigate these costs. For instance, utilizing the post-extraction waste in a biorefinery to produce biofuel or generate electricity can contribute to cost reduction [57]. Thus, with the right strategies and a focus on resource optimization, supercritical fluid extraction technology holds considerable promise for sustainable and economically viable industrial applications.

## 8. Concluding Remarks

This review demonstrated the tremendous potential of critical-fluid-based extraction technologies for the efficient extraction of bioactive chemicals from a variety of natural matrices. There is nevertheless room for development. One potential improvement is linked to a precise understanding of the nature of the component and its quantity, which aids in increasing the extraction process efficiency. Non-SCF extraction processes rely primarily on organic solvents, resulting in waste that must be burnt using fossil fuels, a method that is unsustainable in the long run. Not only is critical fluid extraction a viable alternative approach for collecting valuable chemicals from waste biomass, but it also has the potential to improve the remaining biomass’s downstream processing. Furthermore, the purity of the extract, as well as the time it took to extract it, had a significant impact on the process’s cost and profit. The impact of process parameters on product yield and composition can help in choosing the ideal extraction conditions. These data are critical for optimization, modeling, and scale-up efforts. Extracted chemicals from waste materials can be utilized as food additives or dietary supplements, or they can be employed in the pharmaceutical sector, for example, in molecular imaging in cancer therapy or the manufacturing of antidiabetic medicines such as phloridzin from apples or α-amylase from grapes.

Despite the reported benefits of SCFs in extracting bioactive molecules from agricultural food processing byproducts, several challenges persist. First, optimizing the scalability of this technology and understanding the kinetics of the extraction process is important to enhance bioactive compound yield. Second, a comprehensive evaluation of these molecules’ interaction with food components, their bioavailability, and their primary health benefit mechanisms requires further research. Third, the standard solvent (CO_2_) primarily targets non-polar components; thus, efficient pairing with entrainers for polar substance extraction remains a relevant subject for discussion. Lastly, the fluid’s density limits extraction of macromolecular substances, such as proteins. Consequently, the feasibility of extracting large molecular mass substances using SCF poses a future challenge.

## Figures and Tables

**Table 1:** Usage of valorized extracts in different industries.

Waste Material	Valorized Extract	Usage	Reference
Custard apple seed powder	Volatile and non-volatile components	Flavoring industry	[40]
Apple seed	Linoleic acid and phloridzin, amygdalin absence (antinutrient), higher oxidative stability	Pharmaceutical industry	[10]
Tomato seeds and skins	205 mg per 100 g of lycopene and 75 mg per 100 g of β-carotene	Food and pharmaceutical industries	[16]
Strawberry pomace	46.8 mg/mL saturated FAs, 64.0 mg/mL monounsaturated FAs, and 145.8 mg/mL polyunsaturated FAs	Food and cosmetic industries	[50]
Banana peels	Bioactive compounds; gallic acid, quercetin, and β-carotene	Pharmaceutical industry	[51]
Orange peels	Citronellol, β-pinene, α-pinene, myrcene, terpinolene, C8-aldehyde, linalool, and d-limonene	Pharmaceutical industry	[52]
Sweet potato peels	β-carotene (99.8%), lutein (68.2%), and antioxidant activity (20.7%)	Food and pharmaceutical industries	[15]
Potato peels	Caffeic acid (0.75 mg/g), phenolic recovery 37%, and antioxidant activity 73%	Pharmaceutical and nutraceutical industries	[26]
Onion outer dry layers	Protocatechuic acid mg/100 g and quercetin equivalents	Pharmaceutical industry	[53]
Kiwifruit pomace, skin, and seeds	Phenolic compounds, protocatechuic acid, caffeic acid, catechin	Pharmaceutical industry	[54]
Avocado processing waste	Oleic and linoleic acid, higher phenolic and antioxidant ability as compared to Soxhlet extract	Pharmaceutical industry	[11]
Broccoli processing waste (stems and leaves)	β-carotene, phytosterols, chlorophylls, and phenolic compounds	Cosmetic industry	[18]
Grape pruning waste	Antibacterial and α-amylase inhibition activity	Pharmaceutical industry	[55]
Grape skin, seeds, and pomace	Antioxidants, vitamins, and polyphenols	Pharmaceutical and nutraceutical industries	[19]
Guava seed	Linoleic acid, oleic acid, tocopherol, and phytosterols	Dairy industry	[12]
Mango kernel	Polar lipids 3.38% with desirable phosphorus content	Pharmaceutical and nutraceutical industries	[56]
Tomato cannery waste (peels and seeds)	97% lycopene recovery, spent material showed 64% biodegradability	Food and pharmaceutical industries	[57]
Tomato peels	91% carotenoid recovery in which 96.9% was β-carotene, 87.9% antioxidation	Food and nutraceutical industries	[15]
Peach peels	94.2% total carotenoids of which 75.3% was lutein and 34.1% antioxidation activity	Food and nutraceutical industries	[15]
Grape (*Palomino fino*) pomace	2176 mg/100 g resveratrol of dry sample	Pharmaceutical and nutraceutical industries	[58]
Citrus peels	33 volatile compounds, polymethoxyflavones, limonoids, and phytosterols	Flavoring and pharmaceutical industries	[59]
Apple pomace	Higher antioxidation, 5.63 TEA/g of extract as compared to conventional method	Nutraceutical industry	[20]
Passion fruit bagasse	23.9 g oil/100 g feed, including tocols, carotenoids, and fatty acids	Nutraceutical and cosmetic industries	[17]

**Table 2:** Extraction of valuable bioactive using critical fluids.

Studies Using CO_2_ in Combination with Other Solvents				
Technique	Sample	Treatment Condition	Overall Outcomes	Reference
SFE-CO_2_, 15.5% ethanol as co-solvent	Tomato and peach peels	350 bar, 59 °C, 5 g/min, 30 min	91% and 94.2% carotenoid recovery, with considerable content of β-carotene and lutein	[15]
SFE-CO_2_, 5% ethanol as co-solvent	Apple pomace	30 MPa, 45 °C, 2 L/h, 2 h	Higher antioxidation, 5.63 TEA/g of extract as compared to conventional method	[20]
SFE-CO_2_, 5% ethanol as co-solvent	Grape (*Palomino fino*) pomace	400 bar, 55 °C, 0.8 g/min, 3 h	2176 mg/100 g resveratrol on dry basis	[58]
SFE-CO_2_, ethanol as co-solvent 7%	Broccoli (*Brassica oleracea* var. italica) stem and leaves	443 bar, 40 °C, 31 g/min	β-carotene, chlorophylls, phytosterols, and phenolic compounds	[18]
SC-CO_2_, ethanol as a co-solvent 10%	Tomato (*Lycopersicon esculentum* L.) waste, seeds and skins	150 bar, 20 °C, and 5 mL/min	Lycopene 205 mg per 100 g and β-carotene 75 mg per 100 g of extracted oleoresin	[16]
SFE-CO_2_, ethanol as co-solvent	Grape (*Vitis vinifera* L.) skin, seeds and pomace	250 bar, 60 °C, 2 mL/min	Trans-resveratrol, β-sitosterol, α-tocopherol, and ascorbic acid	[19]
SC-CO_2_ + 1.5:1 ethanol	Avocado (*Persea americana*) seeds and peels	25 MPa, 80 °C	6.9% oil yield, major components were oleic and linoleic acid	[11]
SC-CO_2_, exogenous tomato oil as co-solvent	Tomato solid waste	380 bar, 80 °C and 15 kg/h	97% lycopene recovery	[57]
Studies using CO_2_ and water as a sole solvent				
SFE	Apple (*Malus pumila*) seed	24 MPa, 40 °C, 1 L/h, and 140 min	Linoleic acid (63.76 g/100 g) and phloridzin (2.96 μg/g of seed)	[10]
SC-CO_2_	Strawberry pomace	300 bar, 70 °C, 5 h	26% essential FAs	[50]
SWE	Grape (Tinta Roriz and Touriga Nacional) pruned shoots	40 bars, 150 °C, 40 min	Antimicrobial and enzyme inhibition activity	[55]
SFE-CO_2_	Mango kernel	50 MPa, 40 °C, and 30 g/min	Polar lipid 3.28%, desirable phosphorus content (91.2 mg/kg)	[56]
SFE-CO_2_	Guava (*Psidium guava*) seed	35.7 MPa, 52 °C, 30 g/min and 150 min	Linoleic acid (78.5%), oleic acid (13.8%), phenolics, tocopherol, and phytosterol compounds	[12]
SC-CO_2_	Custard apple (*Annona Squamosa*) seed powder	15 MPa, 308 K, and 1.5 mL/min for volatile and 25 MPa, 318 K, and 2.5 mL/min for nonvolatile components	Volatile and non-volatile components	[40]
SWE	Kiwifruit waste (pomace, skin, and seeds)	50 bar, 200 °C, and 90 min	Phenolic compounds (60.53 mg CaE/g DW), protocatechuic acid, caffeic acid, catechin	[54]
SFE-CO_2_	Passion fruit bagasse	17–26 MPa, 60 °C, 1.80 × 10^−4^ kg/s flow rate	5.8 and 1.5 times more carotenoids and tocols were extracted in sequential process	[17]

## Data Availability

Data is contained within the article.
